# 4-Carbomethoxyl-10-Epigyrosanoldie E Extracted from Cultured Soft Coral *Sinularia sandensis* Induced Apoptosis and Autophagy via ROS and Mitochondrial Dysfunction and ER Stress in Oral Cancer Cells

**DOI:** 10.1155/2022/3017807

**Published:** 2022-10-13

**Authors:** Yun-Ying She, Jen-Jie Lin, Jui-Hsin Su, Ting-Shou Chang, Yu-Jen Wu

**Affiliations:** ^1^Department of Otolaryngology-Head and Neck Surgery, Kaohsiung Veterans General Hospital, Taiwan; ^2^Yu Jun Biotechnology Co., Ltd., Kaohsiung, Taiwan; ^3^Department of Food and Nutrition, Meiho University, Pingtung 91202, Taiwan; ^4^National Museum of Marine Biology and Aquarium, Pingtung 94450, Taiwan

## Abstract

Oral cancer is a malignant neoplasia that is more common in Asian than other regions, and men are at higher risk than women. Currently, clinical treatment for oral cancer consists of radiation therapy combined with chemotherapy. Therefore, it is important to find a drug that can inhibit the growth of cancer cells more effectively and safely. In this study, we examined the cytotoxicity of 4-carbomethoxyl-10-epigyrosanoldie E extracted from cultured soft coral *Sinularia sandensis* towards oral cancer cells. MTT cell proliferation and colony formation assays were used to evaluate cell survival, and immunofluorescence staining and Western blotting were employed to analyze the effects of 4-carbomethoxyl-10-epigyrosanoldie E on apoptosis and autophagy. 4-Carbomethoxyl-10-epigyrosanoldie E treatment also induced the formation of reactive oxygen species (ROS), which are associated with 4-carbomethoxyl-10-epigyrosanoldie E-induced cell death. In addition, the 4-carbomethoxyl-10-epigyrosanoldie E-induced antiproliferation effects on Ca9-22 and Cal-27 cells were associated with the release of cytochrome c from mitochondria, activation of proapoptotic proteins (such as caspase-3/-9, Bax, and Bad), and inhibition of antiapoptotic proteins (Bcl-2, Bcl-xl, and Mcl-1). 4-Carbomethoxyl-10-epigyrosanoldie E treatment also triggered endoplasmic reticulum (ER) stress, leading to activation of the PERK/elF2*α*/ATF4/CHOP apoptotic pathway. Moreover, increased expressions of Beclin-1, Atg3, Atg5, Atg7, Atg12, Atg 16, LC3-I, and LC3-II proteins indicated that 4-carbomethoxyl-10-epigyrosanoldie E triggered autophagy in oral cancer cells. In conclusion, our findings demonstrated that 4-carbomethoxyl-10-epigyrosanoldie E suppressed human oral cancer cell proliferation and should be further investigated with regard to its potential use as a chemotherapy drug for the treatment of human oral cancer.

## 1. Introduction

Oral cancer most often occurs in men over 40 years of age, and more than 95% of cases are derived from the *oral mucosal epithelium* as squamous cell carcinoma, verrucous carcinoma, adenoid cystic carcinoma, or mucinous epidermis-like carcinoma, among which oral squamous cell carcinoma is the most common. The main causes of oral cancer are betel nut-chewing, alcohol consumption, smoking, poor oral hygiene habits, and viral infections [1, 2]. In Taiwan, approximately 90% of oral cancer patients have the habit of chewing betel nut. According to the 2015 report from the Health Promotion Administration, Ministry of Health and Welfare, Taiwan, oral cancer ranked the fifth leading cause of death from malignant tumors in Taiwan [3, 4].

Early treatment of oral cancer can result in a 5-year survival rate of up to 80%, while late diagnosis and treatment can reduce the survival rate to 30% and lead to a high risk of recurrence [5, 6]. Current clinical treatment for oral cancer mostly consists of concurrent chemoradiotherapy (CCRT) [7], and the most commonly used chemotherapeutic drug is 5-Fu. However, side effects caused by 5-Fu include gastrointestinal symptoms such as loss of appetite, stomatitis, vomiting, and diarrhea, in addition to decreased white blood cells and abnormal liver function, which often cause difficulties in treatment. Thus, it is particularly important to identify safe complementary drugs for CCRT [8, 9].

Reactive oxygen species (ROS) are active molecules containing oxygen and are natural by-products of the normal metabolism of oxygen. ROS play an important role in cellular signaling and homeostasis in the body [10]. However, cellular stress caused by changes in the external environment may dramatically increase the concentration of ROS in cells, causing significant damage to the cellular structure and resulting in oxidative damage [11, 12]. Previous studies have shown that apoptosis is accompanied by an increase in the expression of ROS, which causes damage to polyunsaturated fatty acids in the cell membrane and further leads to mutations in the nucleotides of DNA. Therefore, ROS are an indicator of cellular stress of cells in apoptosis [13].

The process of apoptosis can be triggered by either extrinsic or intrinsic pathways in the cell. Intrinsic pathways are triggered by the intracellular signaling pathway, which involves interactions between endoplasmic reticulum (ER) stress and mitochondria [14, 15]. The ER is responsible for protein synthesis and protein folding. When cells are under stress (inflammation, oxidation, and hypoxia), Ca^2+^ homeostasis in the ER is disrupted, resulting in the accumulation of unfolded or misfolded proteins in the inner lumen of the ER, which leads to ER stress. In response to ER stress, cells activate a series of signaling pathways, also referred to as the unfolded protein response (UPR). If the UPR cannot improve the condition of the cells in time, the ER will activate apoptosis, causing self-destruction to reduce stress [16].

Mitochondrial dysfunction has been shown to be a major cause of apoptosis due to dysregulation of Bcl-2 family homeostasis and alteration of the mitochondrial membrane potential, leading to the release of cytochrome *c* from the mitochondrial intermembrane space to the cytosol [17]. Mitochondrial dysfunction promotes a series of reactions, beginning with the activation of caspase-9, which further activates downstream caspase-3; activated caspase-3 then cleaves and activates poly ADP-ribose polymerase 1 (PARP1), which subsequently leads to apoptosis [18, 19].

In addition, autophagy is a cellular metabolic pathway that allows cells to break down their own organelles or other intracellular materials by lysosomes, which is currently considered to be an important mechanism in the regulation of cell growth, differentiation, and cellular and tissue homeostasis. At the end of apoptosis, initiation of autophagy occurs when phagocytes recognize the exposure of phosphatidylserine (PS) on the outer plasma membrane of target cells [20].

Compounds isolated from soft corals have been shown to have the ability to induce apoptosis in cancer cells. In the present study, we analyzed 4-carbomethoxyl-10-epigyrosanoldie E, a compound obtained from soft coral *Sinularia sandensis*, with regard to its effects on the induction of ROS production, mitochondrial dysfunction, and ER stress in oral cancer cells. We further investigated the mechanism of its cytotoxicity leading to apoptosis and autophagy. The results of this study will be important in terms of the potential development of 4-carbomethoxyl-10-epigyrosanoldie E as a new drug for the treatment of cancer.

## 2. Materials and Methods

### 2.1. Materials

Reagents Dulbecco's modified Eagle's medium (DMEM), trypsin-ethylenediaminetetraacetic acid, fetal bovine serum (FBS), and phosphate-buffered saline (PBS) were obtained from Biowest (Nuaillé, France). Polyvinylidene difluoride (PVDF) membranes, goat anti-rabbit, and horseradish peroxidase-conjugated immunoglobulin (Ig) G were obtained from Millipore (Billerica, MA, USA). Protease inhibitor cocktail, DMSO, salubrinal (Sal), 3-(4,5-dimethylthiazol-2-yl)-2,5-diphenyltetrazolium bromide (MTT), ROS Green Flow Cytometry Assay Kit, Z-DEVD-FMK (caspase-3 inhibitor), Z-LEHD-FMK (caspase-9 inhibitor), 3-methyladenine (3-MA), and rabbit anti-human *β*-actin antibodies were obtained from Sigma (St. Louis, MO, USA). Cell extraction RIPA buffer was obtained from TOOLS (Taiwan). Enhanced chemiluminescence (ECL) Western blotting reagents were obtained from Pierce Biotechnology (Rockford, IL, USA). Antibodies against Beclin-1, LC3-I, LC3-II, Atg3, Atg5, Atg7, Atg12, and Atg16 were obtained from Epitomics (Burlingame, CA, USA). Antibodies against procaspase 3, cleaved-caspase 3, procaspase 9, cleaved-caspase 9, cytochrome *c*, Bax, Bad, *p*-Bad, Bcl-2, Bcl-xl, Mcl-1, GRP78, CALR, elF2*α*, *p*-elF2*α*, IRE1*α*, PERK, *p*-PERK, ATF4, ATF6-f, CHOP, and PARP1 were obtained from Cell Signaling Technology (Danvers, MA, USA).

### 2.2. Chemical Structure of 4-Carbomethoxyl-10-Epigyrosanoldie E

**Figure 1:**
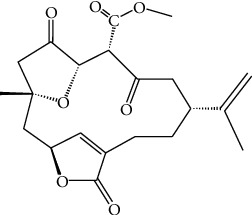
Chemical structure of 4-carbomethoxyl-10-epigyrosanoldie E.

Natural product 4-carbomethoxyl-10-epigyrosanoldie E was isolated from cultured soft coral *Sinularia sandensis* by Dr. Jui-Hsin Su (Figure 1).

### 2.3. Cell Culture

Ca9-22 and Cal-27 oral cancer cells were purchased from the Food Industry Research and Development Institute (Hsinchu, Taiwan) and grown in DMEM, 4 mM L-glutamine, 10% (*v*/*v*) FBS, 100 *μ*g/mL streptomycin, 100 U/mL penicillin, and 1 mM sodium pyruvate in a humidified atmosphere of 5% CO_2_ in air at 37°C.

### 2.4. Cell MTT Assay

Ca9-22 and Cal-27 cells were seeded on 24-well culture plates at a density of 1 × 10^4^ cells/well. After incubation with various concentrations of 4-carbomethoxyl-10-epigyrosanoldie E for 24 hr, 50 *μ*L of MTT solution (1 mg/mL in PBS) were added to each well. Cells treated with DMSO were used as a blank control. The cell culture plates were incubated at 37°C for 4 hr, following which cells were lysed with 200 *μ*L DMSO. The optical density (OD) was measured at 595 nm using a microtiter ELISA reader (Bio-Rad, Hercules, CA, USA). All experiments were repeated three times. We followed the methods of Lin et al. [21].

### 2.5. Clonogenic Assay

Cells were seeded at 1 × 10^2^/well in a 6-well plate. After the addition of 4-carbomethoxyl-10-epigyrosanoldie E at various concentrations to the cells, the cultures were maintained in a 5% CO_2_ incubator at 37°C for 14 days. Thereafter, 0.1% crystal violet was added to stain the cells for 15 minutes, followed by washing three times with PBS. Images of the cells were then obtained using a scanner for subsequent analysis of cell colonies.

### 2.6. DAPI and TUNEL Staining

Cells (1 × 10^5^ cells/well) seeded in a 12-well plate were treated with 10, 15, or 20 *μ*M 4-carbomethoxyl-10-epigyrosanoldie E for 24 hr, and DMSO was used as the control. Cells in each treatment and control group were fixed with 4% paraformaldehyde in PBS solution for 15 min and stained by DAPI according to the manufacturer's instructions. The DeadEnd™ Fluorometric TUNEL System (Promega) was used to detect nuclear DNA fragmentation according to the manufacturer's manual. The cells were photographed under a fluorescence microscope.

### 2.7. Protein Extraction and Estimation

Cells were treated with different concentrations of 4-carbomethoxyl-10-epigyrosanoldie E and incubated for 24 hr, following which a cell extraction buffer was used to accomplish lysis and protein extraction. After centrifugation at 12000 rpm for 10 minutes, Bradford dye (Bio-Rad) was added to the supernatant for protein quantification.

### 2.8. Western Blotting Analysis

The treated samples and control samples (25 *μ*g) were separated by 12.5% SDS-PAGE and transferred onto a PVDF membrane for 1.5 hr at 400 mA using Transphor TE 62 (Hoeffer). The membranes were subsequently incubated with 5% dehydrated skimmed milk in PBS (10 mM NaH_2_PO_4_, 130 mM NaCl) to block nonspecific protein binding, and then incubated with primary antibodies at 4°C overnight. After washing with PBST 5 times (PBS with 0.002% tween 80), the secondary antibody (horseradish peroxidase-conjugated goat anti-rabbit, 1 : 7000 in blocking solution) was added, followed by incubation for 2 hr at 4°C, and finally, visualization was performed using chemiluminesence (Pierce Biotechnology, Rockford, IL, USA). We followed the methods of Lin et al. [21].

### 2.9. ROS Analysis

Cells (1 × 10^5^ cells/well) seeded in a 6-well plate were treated with 5, 10, 15, or 20 *μ*M 4-carbomethoxyl-10-epigyrosanoldie E for 4 hr. Then, 1 *μ*L of 10 *μ*M dichlorofluorescin diacetate (DCFH-DA; Sigma, USA) was added to the wells in the dark, and after mixing and reacting for 15 minutes, changes in the level of H_2_O_2_ in the cells were assessed using a flow cytometer (FACStar Plus) at wavelengths of 488 and 530 nm [22].

### 2.10. Statistical Analysis

Cell viability assay and colony assay data and ROS measurements were collected from three independent experiments and analyzed using Student's *t*-test (Sigma-Stat2.0, San Rafael, CA, USA). Results with *p* < 0.05 were considered statistically significant.

## 3. Results

### 3.1. Effect of 4-Carbomethoxyl-10-Epigyrosanoldie E on Cell Proliferation

**Figure 2:**
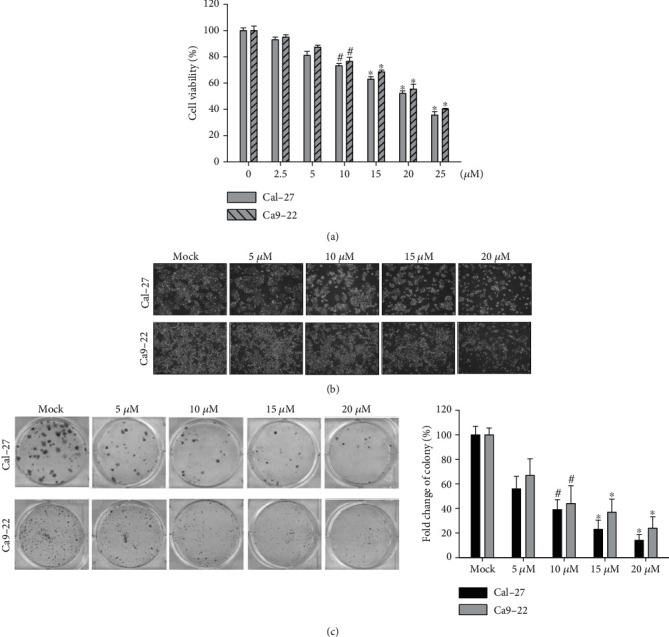
Cytotoxicity and morphology changes conferred by 4-carbomethoxyl-10-epigyrosanoldie E on oral cancer cell lines Ca9-22 and Cal-27. (a) Cytotoxicity of 4-carbomethoxyl-10-epigyrosanoldie E measured using an MTT assay. (b) Cell morphology and growth density in cells treated with different concentrations of 4-carbomethoxyl-10-epigyrosanoldie E. (c) Effect of 4-carbomethoxyl-10-epigyrosanoldie E on colony formation. ^#^*p* < 0.05, ^∗^*p* < 0.001.

An MTT assay was used to evaluate the cytotoxicity of 4-carbomethoxyl-10-epigyrosanoldie E on Ca9-22 and Cal-27 oral cancer cell lines. The results showed that in cells treated with different concentrations of 4-carbomethoxyl-10-epigyrosanoldie E (2.5-25 *μ*M) for 24 hr, a dose-dependent cytotoxicity was observed. When the concentration of 4-carbomethoxyl-10-epigyrosanoldie E was increased to 20 *μ*M, the cell survival rate was still approximately 60% (Figure 2(a)). In order to avoid including cells with a low viability under a high 4-carbomethoxyl-10-epigyrosanoldie E concentration that may undergo cell death not relative to apoptosis, concentrations of 0~20 *μ*M were chosen for use in subsequent experiments.

Cytotoxicity and morphology analyses showed that 4-carbomethoxyl-10-epigyrosanoldie E inhibited cell proliferation in Ca9-22 and Cal-27 cells (Figure 2(b)). In order to verify the results, Ca9-22 and Cal-27 cells were subjected to colony formation analysis with 4-carbomethoxyl-10-epigyrosanoldie E treatment (5, 10, 15, and 20 *μ*M) for 2 weeks. The results showed that cell proliferation was decreased with increased concentrations of 4-carbomethoxyl-10-epigyrosanoldie E (Figure 2(c)).

### 3.2. Apoptosis in Oral Cancer Cells Induced by 4-Carbomethoxyl-10-Epigyrosanoldie E

**Figure 3:**
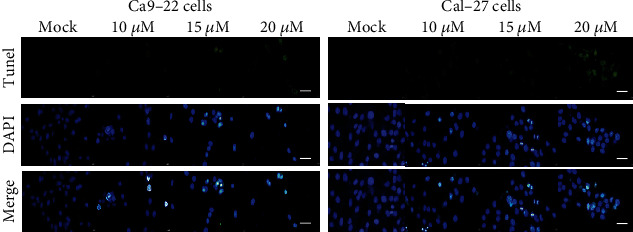
TUNEL/DAPI analysis of apoptosis induced by 4-carbomethoxyl-10-epigyrosanoldie E treatment in Ca9-22 and Cal-27 cells. Ca9-22 and Cal-27 cells were treated with DMSO or 4-carbomethoxyl-10-epigyrosanoldie E at final concentrations of 10, 15, and 20 *μ*M for 24 h. Cells were harvested for DAPI and TUNEL staining as described in “Materials and Methods.” Scale bar = 50 *μ*m.

As the mechanism by which 4-carbomethoxyl-10-epigyrosanoldie E inhibits cell growth has not been studied, we therefore investigated whether 4-carbomethoxyl-10-epigyrosanoldie E causes apoptosis in Ca9-22 and Cal-27 cells using TUNEL/DAPI staining. DNA fragmentation was observed in cells incubated with 4-carbomethoxyl-10-epigyrosanoldie E for 24 hr, and the ratio of DNA fragmentation was increased with increased concentrations of 4-carbomethoxyl-10-epigyrosanoldie E, suggesting that this treatment caused apoptosis in both Ca9-22 and Cal-27 cells (Figure 3).

### 3.3. ROS Production Induced by 4-Carbomethoxyl-10-Epigyrosanoldie E

ROS are highly reactive and may attack molecules in cells to trigger a sequential free-radical reaction. Under normal physiologic conditions, ROS perform normal cellular functions; however, excessive ROS production may cause increased oxidation of DNA, lipids, and proteins, resulting in cellular damage. Evidence suggests that ROS act as secondary messengers, activating transcription factors, and are associated with genes that induce inflammatory responses [23, 24]. In addition, ROS play a role in the mechanism of cell death [25].

**Figure 4:**
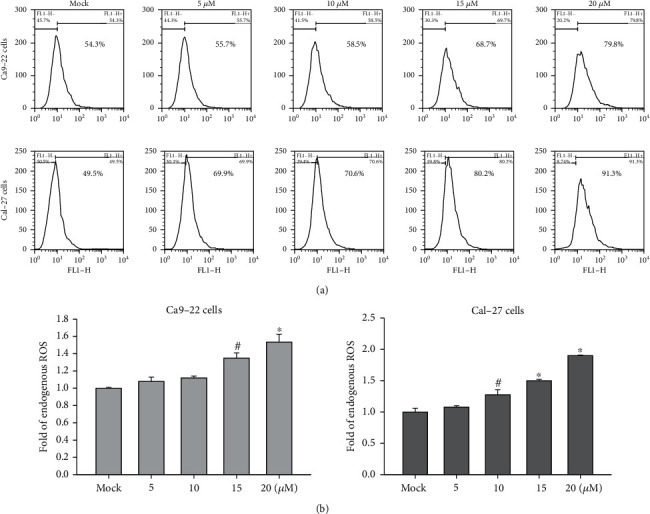
Flow cytometry analysis of ROS changes in oral cancer cells following 4-carbomethoxyl-10-epigyrosanoldie E treatment. (a) ROS levels in Ca9-22 and Cal-27 cells treated with different concentrations of 4-carbomethoxyl-10-epigyrosanoldie E, measured by flow cytometry. (b) Quantification of ROS levels in the experiment groups as compared with the mock control. ^#^*p* < 0.05, ^∗^*p* < 0.001.

ROS may also be involved in early regulation of the apoptotic process, contributing to mitochondrial membrane depolarization, Bax relocalization, cytochrome *c* release, caspase activation, and DNA fragmentation [26, 27]. Therefore, we used flow cytometry to measure the changes in the ROS level in cells treated with 4-carbomethoxyl-10-epigyrosanoldie E and found that ROS production was increased with increased concentrations of 4-carbomethoxyl-10-epigyrosanoldie E. The results indicated that 4-carbomethoxyl-10-epigyrosanoldie E induced ROS production and caused oxidative stress in cells, which in turn promoted the occurrence of apoptosis (Figure 4).

### 3.4. Analysis of the Mechanism of Mitochondrial Dysfunction Induced by 4-Carbomethoxyl-10-Epigyrosanoldie E

**Figure 5:**
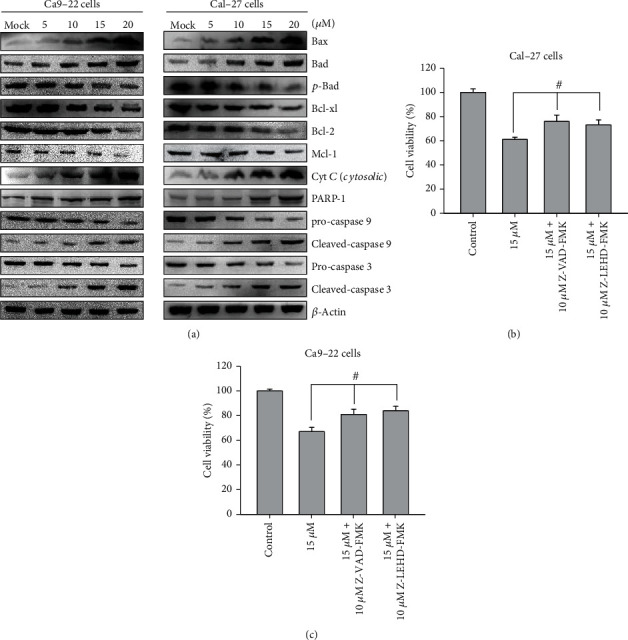
Analysis of the mechanism of apoptosis induced by 4-carbomethoxyl-10-epigyrosanoldie E in Ca9-22 and Cal-27 cells. (a) Western blotting analysis of the expressions of mitochondrial dysfunction-related proteins. (b, c) Suppression of 4-carbomethoxyl-10-epigyrosanoldie E-induced cytotoxicity by caspase-3 and caspase-9 inhibitors. ^#^*p* < 0.05, ^∗^*p* < 0.001.

Western blotting analysis showed that the homeostasis between Bcl-2 and Bax in Ca9-22 and Cal-27 cells was disrupted at 24 hr after 4-carbomethoxyl-10-epigyrosanoldie E treatment. The expressions of antiapoptotic proteins Bcl-2, Bcl-xl, Mcl-1, and *p*-Bad were reduced, while the expressions of proapoptotic proteins Bad and Bax were increased (Figure 5(a)). The level of cytochrome *c* was also increased, and its release into cytosol induced the activation of caspase family proteins. In particular, caspase-3 and caspase-9 are the major players in apoptosis. The increased expressions of cleaved caspase-3 and caspase-9, the activated state of caspases, showed that the apoptotic response was initiated by 4-carbomethoxyl-10-epigyrosanoldie E treatment. The process further caused an increase in the expression of PARP-1, leading to blockage of DNA synthesis and cell death. On the other hand, by adding inhibitors of caspase-3 and caspase-9 (Z-DEVD-FMK and Z-LEHD-FMK), 4-carbomethoxyl-10-epigyrosanoldie E-induced cell death was inhibited, suggesting that caspase-3 and caspase-9 are involved in the mitochondrial dysfunction (Figure 5(b)).

### 3.5. Effect of 4-Carbomethoxyl-10-Epigyrosanoldie E on Apoptosis Induced by Endoplasmic Reticulum Stress

**Figure 6:**
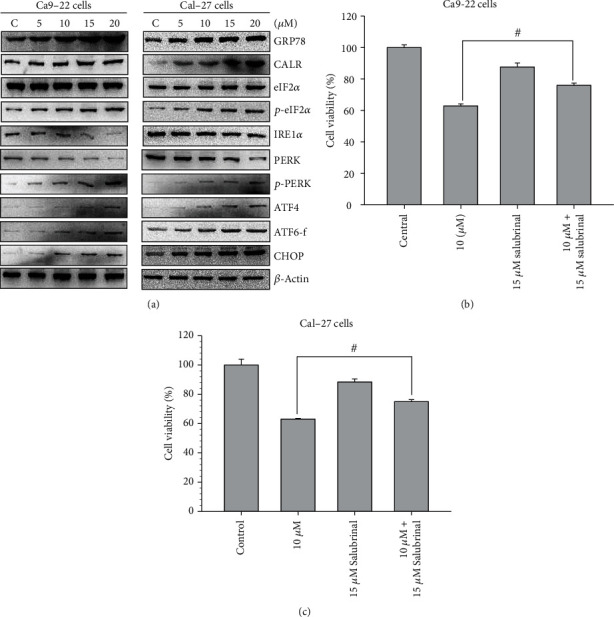
Endoplasmic reticulum PERK/eIF2/ATF4/CHOP pathway in Ca9-22 and Cal-27 cells treated with 4-carbomethoxyl-10-epigyrosanoldie E. (a) Western blotting analysis of ER stress-related proteins. (b, c) Effect of eIF2*α* phosphatase inhibitor salubrinal on 4-carbomethoxyl-10-epigyrosanoldie E-induced apoptosis. ^#^*p* < 0.05, ^∗^*p* < 0.001.

Subsequently, we studied the changes in ER marker proteins and downstream pathways using Western blotting. Calreticulin (CALR) is a molecular chaperone that regulates ER Ca^2+^ homeostasis and binds to misfolded proteins. Alterations in the CALR level may cause ER stress and a UPR, which in turn induce apoptosis [28]. Generation of a UPR will cause GRP78 cleavage and induce the PERK/eIF2*α*/ATF4/CHOP signaling pathway, in addition to triggering the activations of ATF6 and IRE1*α* (the other two main proximal effectors of the UPR), synergistically initiating downstream signaling pathways [29]. Our results demonstrated that 4-carbomethoxyl-10-epigyrosanoldie E increased the expression of CALR in the cells, leading to an increased expression of GRP78, which further upregulated the expressions of downstream molecules *p*-PERK, *p*-eIF2*α*, and ATF4. Increases in the expressions of IRE1*α* and ATF6 increased the CHOP level to induce apoptosis in the cells. The results suggested that the apoptotic mechanism is generated via the PERK/eIF2*α*/ATF4/CHOP signaling pathway in the ER (Figure 6(a)).

We also utilized an inhibitor of eIF2*α* phosphatase, salubrinal, to validate the involvement of the signaling pathway in 4-carbomethoxyl-10-epigyrosanoldie E-induced apoptosis. As shown in Figure 6(b), the results of an MMT assay revealed that salubrinal reduced the apoptosis caused by 4-carbomethoxyl-10-epigyrosanoldie E, indicating that ER stress occurred in the cells treated with 4-carbomethoxyl-10-epigyrosanoldie E.

### 3.6. Auotophagy Caused by 4-Carbomethoxyl-10-Epigyrosanoldie E

**Figure 7:**
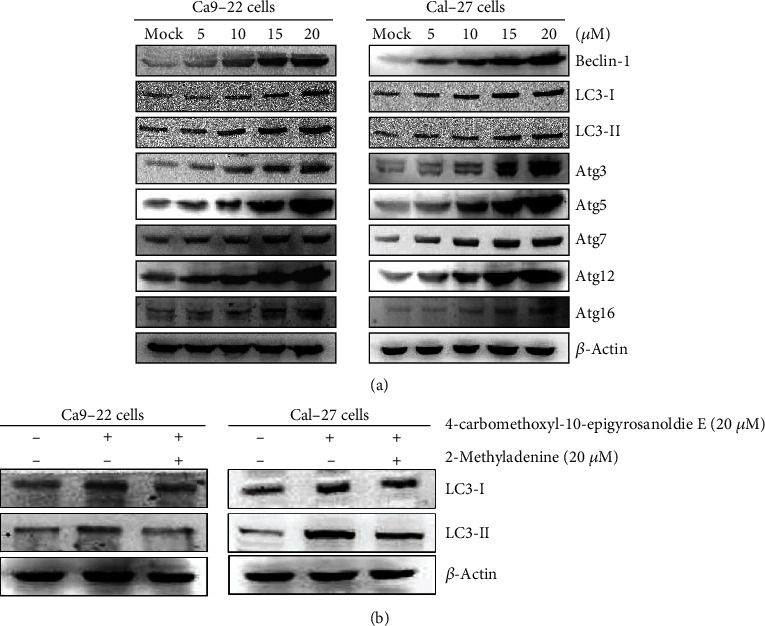
Western blotting analysis of autophagy-related proteins in Ca9-22 and Cal-27 cells treated with 4-carbomethoxyl-10-epigyrosanoldie E. (a) Associated proteins, including Beclin-1, LC3-I, LC3-II, Atg3, Atg5, Atg7, Atg12, and Atg16, were analyzed using Western blotting analysis. (b) The effects of 3-MA application (autophagic inhibitor; 20 *μ*M) prior to treatment with 4-carbomethoxyl-10-epigyrosanoldie E for 24 h on were LC3-I and LC3-II assessed.

Several studies have shown that natural compounds isolated from plants may cause cell death in cancer cells through autophagy [30, 31]. In the present study, we found that the protein expressions of Beclin-1, Atg3, Atg7, Atg5, Atg12, and Atg16 in Ca9-22 and Cal-27 cells were increased with increased concentrations of 4-carbomethoxyl-10-epigyrosanoldie E, and the levels of LC3-I and LC3-II, the main structural proteins of autophagosomal membranes, were also increased (Figure 7(a)). Additionally, when autophagic inhibitor 3-methyladenine (3-MA) was employed, suppression of LC3-I and LC3-II expressions was observed (Figure 7(b)). It can be inferred from these findings that 4-carbomethoxyl-10-epigyrosanoldie E may promote the occurrence of autophagy in oral cancer cells.

## 4. Discussion

The search for effective drugs obtained from natural products has long been the goal of many scientists. Many effective drugs have been developed in the past from natural products, such as Oncovin, Taxol, Navelbine, and Vumon, and some water-soluble camptothecin analogues (e.g., Hycamtin) [32]. Previous studies have shown that compounds extracted from soft corals exert anticancer activities, causing cytotoxicity towards cells via the regulation of multiple cell signaling pathways. Previously, we discovered that 11-*epi*-sinulariolide acetate from soft coral *Sinularia flexibilis* exerts a cytotoxic effect on hepatocellular carcinoma cells through ER stress and mitochondrial dysfunction [33]. In this study, we found that 4-carbomethoxyl-10-epigyrosanoldie E isolated from *Sinularia sandensis* possesses the ability to induce apoptosis in oral cancer cells and revealed the mechanisms involved.

Apoptosis is a programmed cell death triggered by a series of changes in external factors (i.e., extracellular stimuli) or internal signals (e.g., DNA damage, ER stress, and increased ROS level). Our results showed that 4-carbomethoxyl-10-epigyrosanoldie E at 20 *μ*M inhibited cell growth of Ca9-22 and Cal-27 cells by approximately 40% after 24 hr of treatment (Figure 2), and both cell density and morphology were affected by the treatment. TUNEL/DAPI staining showed that higher concentrations of 4-carbomethoxyl-10-epigyrosanoldie E resulted in increased fluorescence signals due to nucleic acid breakage, indicating that 4-carbomethoxyl-10-epigyrosanoldie E induced apoptosis in the cells (Figure 3).

Oxygen is an important molecule for living organisms. By accepting free electrons, it generates reactive oxygen molecules, named ROS or free radicals [34, 35]. ROS are molecules with unpaired electrons, which are unstable free radicals seeking other atoms or molecules to bond with. In the process, they will attack important molecules in cells, including nucleic acids, proteins, and enzymes [36]. In living organisms, oxygen molecules undergo transfer of electrons to produce superoxide anions, which form hydrogen peroxide and then hydroxyl radicals. If the excess production of free radicals cannot be relieved in time, oxidative stress can lead to downstream apoptosis in cells [37, 38]. Therefore, in order to verify that intracellular stress existed in the cells, we used flow cytometry to examine the changes in the levels of ROS in oral cancer cell lines in response to 4-carbomethoxyl-10-epigyrosanoldie E treatment. We found that the ROS level increased with an increasing 4-carbomethoxyl-10-epigyrosanoldie E concentration (Figure 4), suggesting that 4-carbomethoxyl-10-epigyrosanoldie E induces downstream apoptotic signals through inducing oxidative stress in oral cancer cells.

Apoptosis occurs normally in cells during development and aging and may represent a new therapeutic strategy to inform the development of cancer treatment. Apoptosis is regulated by a proteolytic system involving caspase family proteins. Upon receipt of signals of cell death, activation of procaspases results in activated caspases and triggers a series of caspase downstream reactions, which in turn trigger downstream enzymes to damage DNA and cause cell death. Caspases are classified into initiator caspases and executioner caspases; the former include caspase-8, caspase-9, and casaspse-10, which can trigger proteolysis, followed by activation of executioners caspase-3, caspase-6, and caspase-7. Activation of executioner caspases results in a series of protein degradation reactions that lead to apoptotic events [39]. Bcl-2 family proteins have the function to regulate apoptosis and can be classified into three groups: multidomain effectors (e.g., Bax, Bak), BH3-only proteins (e.g., Bad, Bid, and PUMA), and antiapoptotic proteins (e.g., Bcl-2, Bcl-xl, and Mcl-1) [40, 41]. The two main pathways triggered in apoptosis are extrinsic and intrinsic pathways [42, 43]. Induction of the intrinsic pathway occurs when intracellular stress is present in organelles, including mitochondria and the ER. Studies have indicated that mitochondrial dysfunction plays an important role in apoptosis. Bax activation is the key in mitochondria-dependent apoptosis. Under stress conditions, Bax enters the outer membrane of mitochondria, and the permeability of the mitochondrial membrane increases with the formation of nonselective pores in the inner membrane, causing a loss of mitochondrial membrane potential (ΔΨ*m*) [44]. Additionally, an increase in the ratio of Bax/Bcl-2 stimulates the release of cytochrome *c* and AIF proteins from the inner membrane of the mitochondria into the cytosol, which causes cytochrome *c* to bind to ARAF-1 and activate caspase-3. Activated caspase-3 then cleaves its receptor poly (ADP-ribose) and polymerase 1 (PARP-1), causing apoptotic events such as chromosome condensation and DNA breakage [45]. In the present study, Western blotting analysis showed that increased concentrations of 4-carbomethoxyl-10-epigyrosanoldie E resulted in increasing disruption of the homeostasis between Bcl-2 and Bax in Ca9-22 and Cal-27 cells, which led to increases in the expressions of proapoptotic Bcl-2 family proteins Bad and Bax and inhibition of anti-apoptotic proteins Bcl2, Bcl-xl, *p*-Bad, and Mcl-1. During the process, the expressions of cytochrome *c* (cytosolic), activated caspase-3 and caspase-9 (cleaved caspase-3 and 9), and PARP-1 were also increased (Figure 5(a)). The aforementioned results indicated that alteration of mitochondrial activity regulates the occurrence of apoptosis in the cells. Moreover, the addition of caspase-3 and caspase-9 inhibitors increased the cell survival rate (Figure 5(b)), which further suggested that the apoptotic effect of 4-carbomethoxyl-10-epigyrosanoldie E on Ca9-22 and Cal-27 cells is regulated by mitochondrial dysfunction and caspase activation.

The ER is a specialized organelle that is extremely sensitive to environmental stress. When cells are under stress, the ER triggers a stress-related response to evade immediate cell dysfunction. The key step in protein maturation occurs in the ER, where proteins are modified to form their proper tertiary structure by ER-resident enzymes and chaperones. Cellular stress induced by the abnormal accumulation of unfolded or misfolded proteins in the ER, referred to as the UPR, may result in cell damage [46]. The UPR is a cellular response to remove cellular stress and can therefore be considered as a mitigation action by the cell prior to apoptosis, which if not eliminated can lead to cell death [47, 48]. We found that the increase in CALR after 4-carbomethoxyl-10-epigyrosanoldie E treatment led to an increase in the Ca^2^+ concentration in the cytosol and triggered ER stress; this then induced the expression of GRP78, which further triggered the downstream pathway that increased the expressions of *p*-PERK, *p*-eIF2*α*, and ATF4 in a dose-response manner related to the concentration of 4-carbomethoxyl-10-epigyrosanoldie E. In addition, alterations of IRE1*α* and ATF6 and an increasing trend in CHOP were observed, suggesting that this pathway is involved in 4-carbomethoxyl-10-epigyrosanoldie E-induced apoptosis (Figure 6(a)). We also observed that salubrinal rescued cell survival in cells treated with 4-carbomethoxyl-10-epigyrosanoldie E alone (Figure 6(b)), which confirmed the hypothesis that 4-carbomethoxyl-10-epigyrosanoldie E initiated ER stress and consequently induced apoptosis in oral cancer cells. These results indicated that 4-carbomethoxyl-10-epigyrosanoldie E can induce apoptosis via the PERK/eIF2*α*/ATF4/CHOP delivery pathway.

Autophagy is an evolutionary conserved catabolic process that eliminates damaged organelles, misfolded proteins, and invading organisms. Previous studies have suggested that autophagy is also related to cellular stress adaptation, eliminating aging cells under normal conditions and helping to inhibit inflammatory responses under specific cellular stresses. This process is regulated by a variety of lipids and autophagy proteins (Atg), which eventually accumulate into autophagosomes and carry the materials to be broken down to lysosomes, where they are broken down to form amino acids and other substances. Therefore, the proteins involved in autophagy determine the efficiency of the overall cellular metabolism, and may represent an index of cellular response to stress-induced inflammation [49, 50]. The results of the current study showed that the expression of Beclin-1 in the cells increased with an increasing concentration of 4-carbomethoxyl-10-epigyrosanoldie E, indicating that the autophagosome precursors in the initial stage were activated. Therefore, the expressions of Atg3, Atg7, Atg5, Atg12, and Atg16 were also increased, which led to amplified expressions of LC3-I an LC3-II. Moreover, the addition of autophagy inhibitor 3-MA reduced the 4-carbomethoxyl-10-epigyrosanoldie E-induced cytotoxicity and the amounts of LC3-I and LC3-II in Ca9-22 and Cal-27 cells (Figure 7). As a result, we can confirm that 4-carbomethoxyl-10-epigyrosanoldie E induces autophagosomes in oral cancer cells, eventually leading to autophagy.

## 5. Conclusion

4-Carbomethoxyl-10-epigyrosanoldie E, a compound isolated from soft coral *Sinularia sandensis*, was found to induce ROS production in oral cancer cells, leading to the initiation of multiple cellular pathways; these include the apoptotic pathway triggered by ER stress and mitochondrial dysfunction and ultimately cause initiation of autophagy. Our results confirmed that 4-carbomethoxyl-10-epigyrosanoldie E has an excellent activity in inhibiting oral cancer cells and may have the potential to be developed as an effective anticancer drug for the treatment of oral cancers.

## Data Availability

Data generated or analyzed during this study are provided in full within the published article.
